# Differential gene regulatory pathways and co-expression networks associated with fire blight infection in apple (*Malus* × *domestica*)

**DOI:** 10.1038/s41438-019-0120-z

**Published:** 2019-04-06

**Authors:** Katchen Julliany Pereira Silva, Jugpreet Singh, Ryland Bednarek, Zhangjun Fei, Awais Khan

**Affiliations:** 1 Plant Pathology and Plant-Microbe Biology Section, Cornell University, Geneva, NY 14456 USA; 2 Boyce Thompson Institute, Cornell University, Ithaca, NY 14853 USA

**Keywords:** Plant stress responses, Plant sciences

## Abstract

Apple cultivars with durable resistance are needed for sustainable management of fire blight, the most destructive bacterial disease of apples. Although studies have identified genetic resistance to fire blight in both wild species and cultivated apples, more research is needed to understand the molecular mechanisms underlying host–pathogen interaction and differential genotypic responses to fire blight infection. We have analyzed phenotypic and transcriptional responses of ‘Empire’ and ‘Gala’ apple cultivars to fire blight by infecting them with a highly aggressive *E. amylovora* strain. Disease progress, based on the percentage of visual shoot necrosis, started showing significant (*p* < 0.001) differences between ‘Empire’ and ‘Gala’ 4 days after infection (dai). ‘Empire’ seems to slow down bacterial progress more rapidly after this point. We further compared transcriptome profiles of ‘Empire’ and ‘Gala’ at three different time points after fire blight infection. More genes showed differential expression in ‘Gala’ at earlier stages, but the number of differentially expressed genes increased in ‘Empire’ at 3 dai. Functional classes related to defense, cell cycle, response to stress, and biotic stress were identified and a few co-expression gene networks showed particular enrichment for plant defense and abiotic stress response genes. Several of these genes also co-localized in previously identified quantitative trait locus regions for fire blight resistance on linkage groups 7 and 12, and can serve as functional candidates for future research. These results highlight different molecular mechanisms for pathogen perception and control in two apple cultivars and will contribute toward better understanding of *E. amylovora-Malus* pathosystem.

## Disease resistance: How apples fight blight

Different apple varieties, such as ‘Gala’ and ‘Empire,’ defend themselves against fire blight in different ways, and studying how they do it may help in breeding varieties with better resistance. Fire blight is a devastating bacterial disease that can destroy entire orchard blocks in a single season. Breeding for increased resistance is one of the most efficient ways to combat it. Some apple varieties are naturally more resistant than others, but the underlying genetics are not well understood. Awais Khan at Cornell University in New York and co-workers investigated how the ‘Gala’ and ‘Empire’ varieties defend themselves in the first 72 h after infection. ‘Empire’ showed stronger resistance than ‘Gala,’ with many distinct resistance mechanisms. The researchers identified several resistance genes in each variety, which may eventually be used in breeding more blight-resistant apple varieties.

## Introduction

The Gram-negative bacterium *Erwinia amylovora* (Burrill), causative agent of fire blight, represents a major threat to apple (*Malus* × *domestica* Borkh.) production worldwide. Fire blight control mostly relies on preventive measures, including pruning out diseased plant parts, and application of chemicals like copper compounds and bio-control agents^1^. However, bacterial infection can still spread through contaminated orchard management tools, or by the antibiotic resistance mechanisms in some bacterial strains^2^. Bacterial invasion of a susceptible host can cause severe outbreaks under warm temperatures, rain and high humidity^1,3,4^. The most sustainable option for fire blight control is the use of cultivars with durable resistance^5^. Wild *Malus* species and cultivated apples display varying responses to fire blight under specific environments through complex host-pathogen interactions^6,7^. Identification of several strain-specific quantitative trait loci (QTL) linked to fire blight resistance suggests the presence of distinct genetic mechanisms in host plants to respond against bacterial infections^3,7–13^. Identification and characterization of biological processes and gene networks that respond to fire blight infection can improve understanding of host–pathogen interaction and to develop cultivars with improved fire blight resistance.

*E. amylovora* uses *Hrp* T3SS (Hypersensitive reaction and pathogenicity, Type III secretion systems) to deliver virulence-associated molecules directly into the cytosol of host plant cells where they interact with the disease-specific protein, *DspA*/E, for successful fire blight infection in susceptible plants^5,14^. This elicits a hypersensitive response in resistant plants. The exopolysaccharides (EPS) amylovoran and levan thus synthesized are major components required by *E. amylovora* for pathogenicity, virulence, and biofilm formation for survival, colonization, and movement in the host^15,16^. In parallel, plants have developed an arsenal of defenses against fire blight infection. First, *E. amylovora* must overcome several physical barriers on the plant surface, including wax layers, rigid cell walls, cuticular lipids, trichomes, and antimicrobial enzymes or secondary metabolites^15^. In addition, the pathogen signatures are recognized at a molecular level, triggering the upregulation of defense responses such as accumulation of pathogenesis-related (PR) proteins. Systemic acquired resistance (SAR) protects plants from secondary infection by activating multiple signaling pathways and expressing key genes, such as *NPR1, NPR2*, and *NPR10* ^17,18^ to combat infection. Photosynthesis, general metabolism, defense, and signaling pathway genes change expression in apple (*Malus* × *domestica*) during its interaction with *E. amylovora*^6,19–26^. Although these findings have provided important insights into host–pathogen interaction, more studies are required to understand the molecular mechanisms that control differential genotypic responses to fire blight infection.

Fire blight resistance in apples has been mainly studied through quantitative genetic approaches in diverse genetic mapping populations, which has led to the identification of major effect QTLs^5,7,10,15,27–30^. Major QTL for fire blight resistance on linkage group (LG) 3, 10, and 12 were identified from wild Malus species including *Malus robusta, Malus arnoldiana, Malus fusca, Malus evereste*^15^. A few moderate to minor QTLs were also identified from *Malus* × *domestica*, for example, Fiesta^10^, Florina^11^, a PRI breeding line^29^ and Enterprise^31^. Cultivated apples are generally susceptible to fire blight with a few exceptions that have moderate resistance/tolerance (for example, ‘Red Delicious’ and ‘Splendor’). Novel minor resistance alleles from domesticated apples can require less time and fewer cycles of backcrossing to develop breeding lines with improved fire blight resistance. Furthermore, high-throughput RNA-sequencing (RNA-Seq) have been used to characterize molecular mechanisms related to fire blight infection in apple cultivars ‘Golden Delicious’, ‘Idared’ and ‘Free Redstar’^32,33^. RNA-Seq was used to identify transcriptome level changes in flowers of ‘Golden Delicious’ challenged with *E. amylovora* strain CFBP 1430^32^. Also, the transcriptome response of a low-virulence *E. amylovora* strain infecting resistant and susceptible apple cultivars has been studied^33^. The results of these studies highlighted the potential role of genes related to jasmonic acid, ethylene, peroxidase superfamily, phenylpropanoid pathway, pathogenesis-related, and disease resistance in response to fire blight infection^32^. However, the response of apples to fire blight varies significantly with respect to plant tissue being infected, strain virulence, and genetic background of host cultivars^33,34^. It is very likely that different apple cultivars deploy distinct molecular mechanisms to overcome infection from bacterial strains with varying levels of virulence. In addition, time-course analysis of transcriptional changes after bacterial infection and co-expression of different fire blight responsive genes can better define the molecular mechanisms and pathways involved in pathogen perception and control.

Although previous investigations have evaluated the gene expression differences against fire blight infection^32,33^, a rigorous time-course analysis of cultivar-specific responses has not been conducted. We tested the hypothesis that unique molecular pathways dictate the differential host susceptibility to fire blight infection. Phenotypic characterization showed a clearly different response in two apple cultivars, ‘Empire’ and ‘Gala’, to a highly aggressive *E. amylovora* strain^27,34^. Comparison of transcriptome patterns between these two cultivars over time led to identification of potential genes and functional classes involved in disease susceptibility and biotic stress response. We have further highlighted the co-expressed gene networks related to disease response and susceptibility. The results presented in this research will improve the understanding of molecular responses to fire blight disease in apple.

## Results

### Disease assessment

**Fig. 1:**
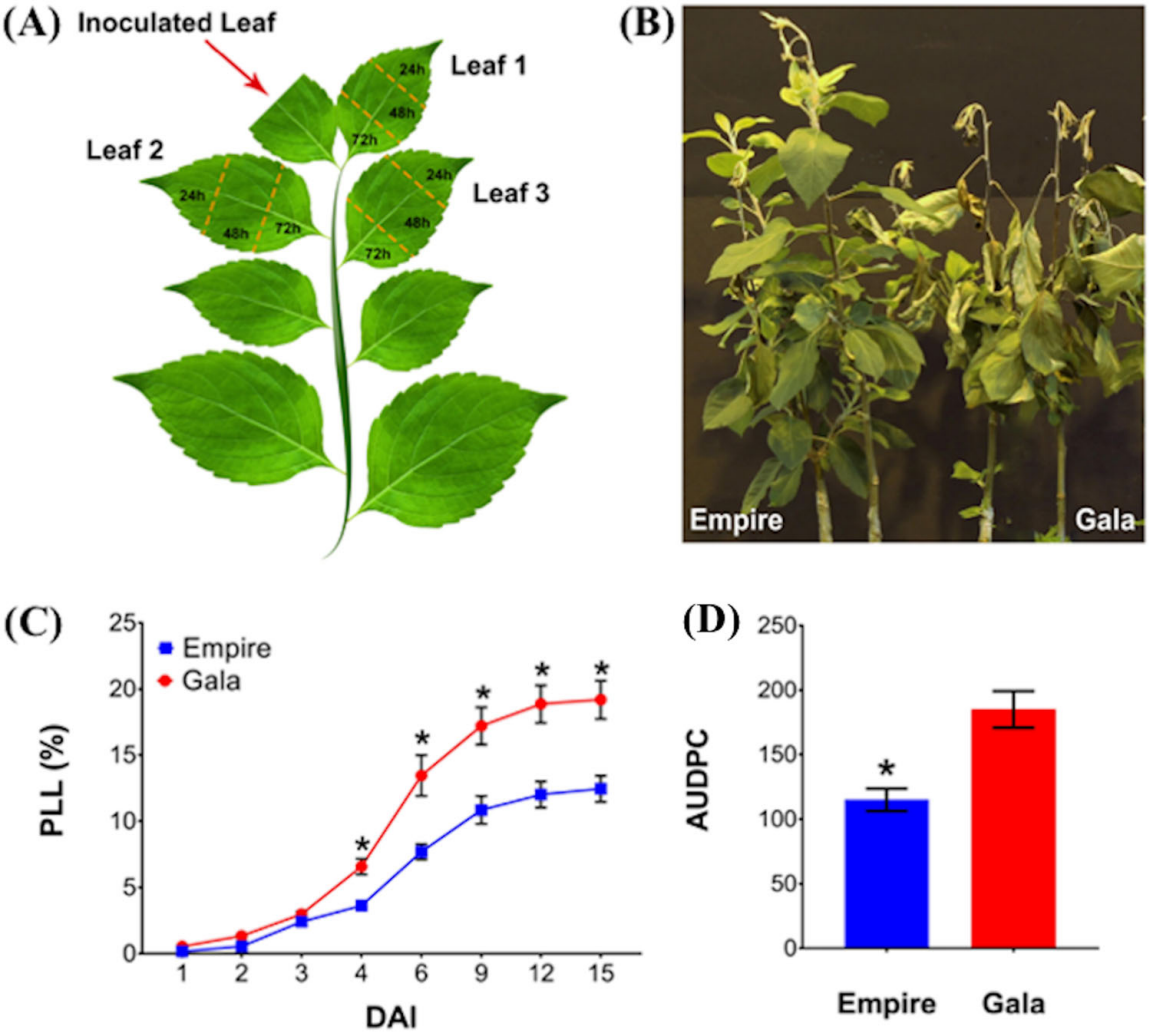
Sampling method, symptoms and disease severity in ‘Empire’ and ‘Gala’ apple cultivars after fire blight infection. **a** Schematic representation of shoot fire blight inoculation and leaf sampling. Inoculation was performed by bisecting the youngest unfolded leaf. One strip (~1 cm in width) from each of the three expanded leaves located immediately below the inoculated leaf were pooled together, to obtain enough tissue for RNA-Seq library construction, at 24, 48, and 72 h post inoculation, respectively; **b** photograph showing example of ‘Empire’ and ‘Gala’ apple cultivars at 15 days after infection with *Erwinia amylovora* strain *Ea2002A*. Grafted plants were 7 months old and infected using scissor inoculation method; **c** fire blight severity in ‘Empire’ and ‘Gala’ apple cultivars. Disease severity is expressed in percentage of lesion length (PLL) of the shoot at 1, 2, 3, 4, 6, 9, 12, and 15 days after infection; **d** area under disease progress curve (AUDPC), 15 days after infection. Data are presented as mean ± SEM. * *p*-value < 0.001, using Student’s *t*-test

Disease evaluation at different time points post *E. amylovora* inoculation (Fig. 1a, b) indicated a clear phenotypic difference between ‘Empire’ and ‘Gala’. Analysis of shoot necrosis measured as percent lesion length (PLL) and area under disease progress curve (AUDPC) showed significant (*p* < 0.001) differences between ‘Empire’ and ‘Gala’ (Fig. 1c, d). Shoot blight was first prominent 3 days after infection (dai) or 72 h after infection (hpi) in both cultivars and presented similar PLL, but at 4 dai, ‘Empire’ showed significantly (*p* < 0.001) less PLL (3.63 ± 0.27%) than ‘Gala’ (6.58 ± 0.59%). AUDPC values calculated at 15 dai were 115 and 185 for ‘Empire’ and ‘Gala’, respectively (Fig. 1d). At 15 dai, the average PLL and AUDPC were significantly lower, 35% and 38%, in ‘Empire’ (*p* < 0.001) (Fig. 1c, d), proving ‘Gala’ as the more susceptible cultivar. Visual detection of light brown to reddish discoloration and bacterial ooze from the point of inoculation were also observed at 72 hpi after infection in both cultivars.

### Global transcriptome patterns related to fire blight infection

**Fig. 2:**
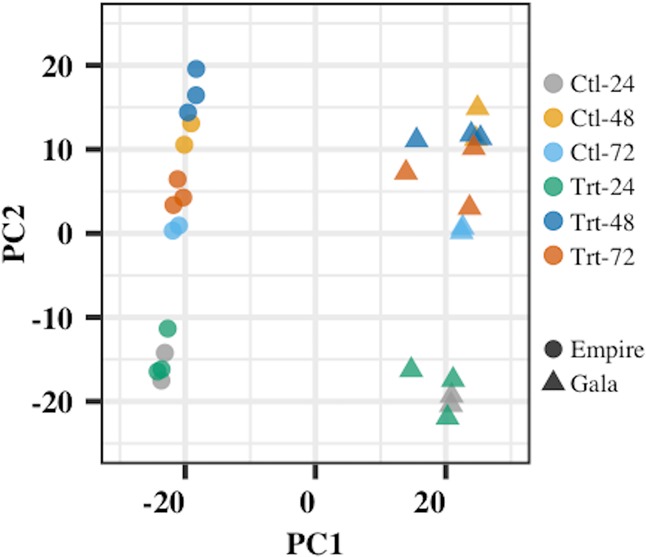
Principal component analysis (PCA) of the log2-transformed normalized gene expression values between different control and treatment samples from ‘Empire’ and ‘Gala’ apple cultivars. Ctl-24 = Control 24 hpi, Ctl-48 = Control 48 hpi, Ctl-72 = Control 72 hpi, Trt-24 = Treatment 24 hpi, Trt-48 = Treatment 48 hpi, Trt-72 = Treatment 72 hpi. hpi hours post inoculation

RNA sequencing yielded approximately 467 million raw reads with a total of 12–89 million reads per treatment (Supporting file S1). Average read count for each treatment ranged from approximately 6 to 23 million. Barcode removal and quality filtering removed around 5% of the raw reads. The resulting high-quality-filtered reads were aligned against the latest apple reference genome GDDH13 v1.1^34^, with mapping rates ranging from 94% to 95.2% (Supporting file S1). Sample clustering with principal component analysis (PCA) showed co-localization of biological replicates for each cultivar, indicating the consistency in sampling and experimental procedure (Fig. 2). This analysis further pointed to cultivar- and treatment-specific effects on the overall patterns of gene expression. For example, the biplot between first two principal components showed a clear distinction between ‘Empire’ and ‘Gala’ samples (Fig. 2). Also, the samples from individual treatments showed clear separation from each other. Differences in gene expression patterns can most likely explain the sample- and genotype-specific clustering in the fire blight transcriptome dataset.

### Differential gene expression and GO enrichment

**Fig. 3:**
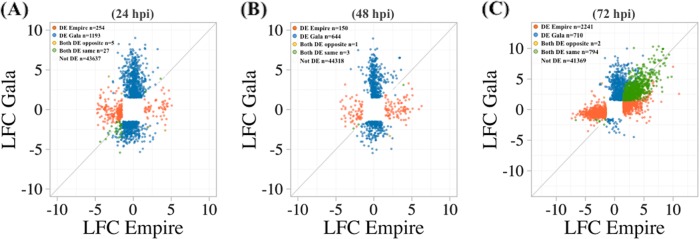
Unique and conserved gene expression response to fire blight infection in ‘Empire’ and ‘Gala’ apple cultivars at. **a** 24 hpi, **b** 48 hpi, and **c** 72 hpi. DEGs from each control vs. treatment comparison were identified and log2 fold values were compared between ‘Empire’ and ‘Gala’ for each time point. LFC log2 fold change, hpi hours post inoculation

Both cultivars exhibited contrasting patterns of gene expression at different time points. ‘Empire’ displayed a larger number of both upregulated and downregulated genes at 72 hpi, whereas Gala had more differentially expressed genes (DEGs) at 24 and 48 hpi (Fig. S1). Empire had total 3037 differentially expressed genes (DEGs) at 72 hpi, out of which 55.4% showed upregulation and 44.6% were downregulated. A completely different expression trend was displayed by ‘Gala’ where 97.1% and 2.9% genes showed up- and downregulation at 72 hpi. A comparison of all the DEGs at three time points indicated that a total of 4622 and 3923 unique genes were up- and downregulated in ‘Empire’, and 3527 and 1398 unique genes were up- and downregulated ‘Gala’ in response to fire blight infection, respectively. A closer analysis of gene expression differences showed only 0.5–2.1% common DEGs between ‘Gala’ and ‘Empire’, indicating that a different set of genes was activated at 24 and 48 hpi treatments for each apple cultivar (Fig. 3a, b, Fig. S2). The percentage of shared genes between the two cultivars was 21.2% at 72 hpi (Fig. 3c, Fig. S2). These results were also observed by comparing the up- and downregulated genes in ‘Empire’ and ‘Gala’ at individual time points (Fig. S2). Shared genes were mainly expressed in the same direction, *i.e*. up- or downregulated in both ‘Gala’ and ‘Empire’ (Fig. 3a–c). The latter type of DEGs ranged from 75% to 99.7% of the shared genes between ‘Empire’ and ‘Gala’ at three time points, whereas DEGs having opposite expression in ‘Gala’ and ‘Empire’ represented only 1–5 at 48 and 24 hpi, respectively (Fig. 3a–c). Low percentage of shared DEGs between ‘Empire’ and ‘Gala’ at each time point further supports the PCA results, indicating that the two cultivars have unique molecular mechanisms against the disease.

At 24 hpi, a total of 62 and 727, and 224 and 498 DE genes were up- or downregulated in ‘Empire’ and ‘Gala’, respectively. Of these, three upregulated genes (MD09G1063300, MD11G1078800, and MD09G1162800) and 23 downregulated genes were shared between the two cultivars, and two (MD13G1224800 and MD05G1197600) were upregulated in ‘Empire’ but downregulated in ‘Gala’. At 48 hpi, the number of upregulated genes (88 and 420) increased and downregulated genes (66 and 228) decreased in ‘Empire’ and ‘Gala’, respectively. Only one upregulated gene (MD09G1142300) and two downregulated genes (MD14G1116200 and MD07G1227800) were common between cultivars, and one (MD17G1187100) was upregulated in ‘Empire’ but downregulated in ‘Gala’ (Fig. S3). The number of DEGs increased significantly at 72 hpi. Total upregulated genes were 1680 and 1461 and downregulated genes were 1357 and 45 in ‘Empire’ and ‘Gala’, respectively. At this time point, ‘Empire’ and ‘Gala’ shared 787 upregulated genes but only 7 were downregulated. Interestingly, ‘Empire’ and ‘Gala’ did not share genes displaying divergent expression patterns at 72 hpi (Fig. S3).

### System-level functional pathways in apple in response to fire blight infection

**Fig. 4:**
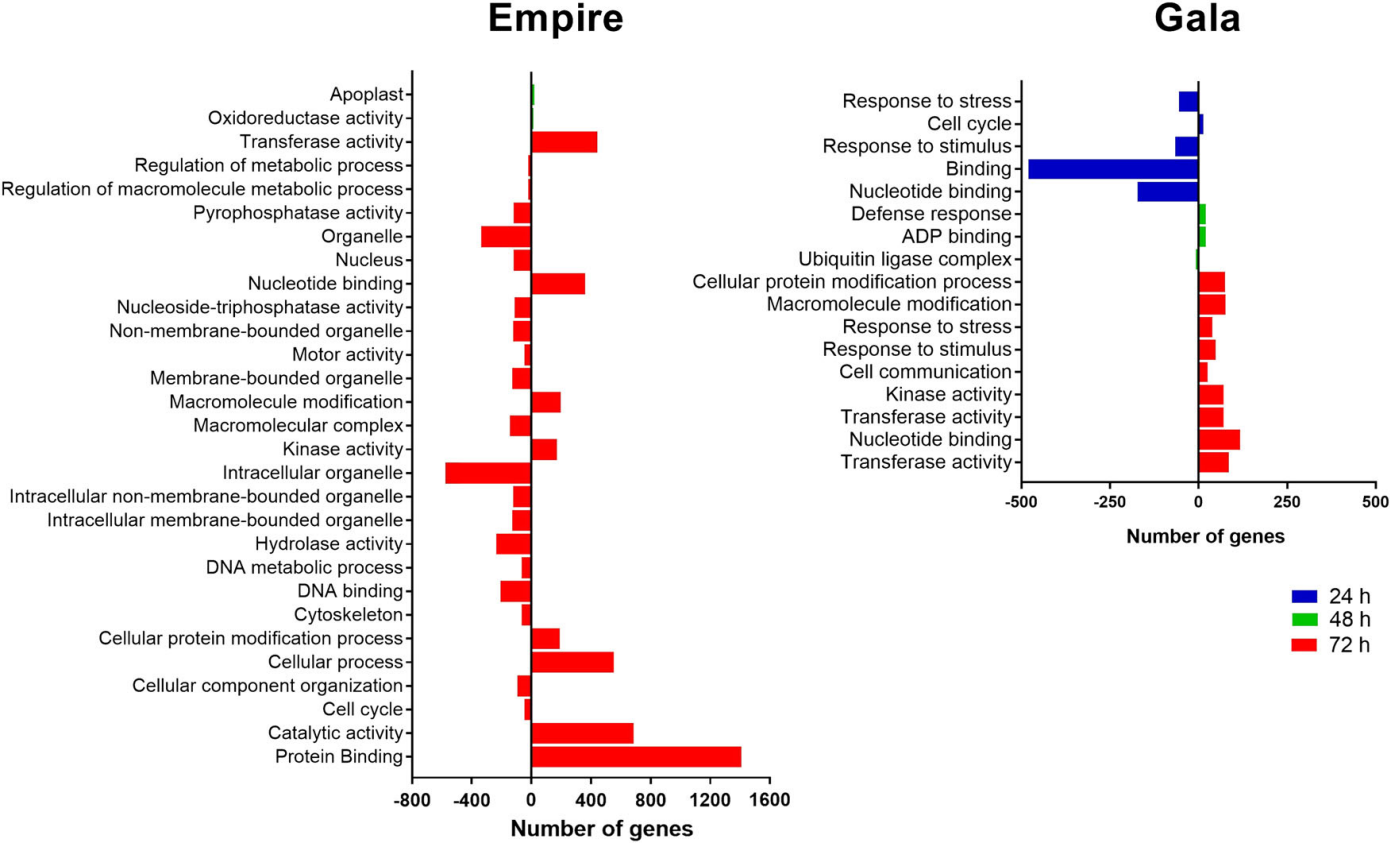
Summary of gene ontology (GO) enrichment analysis of differentially expressed genes after fire blight infection in apple cultivars ‘Empire’ (**a**) and ‘Gala’ (**b**) after 24, 48, and 72 hpi. hpi hours post inoculation

Gene ontology (GO) term enrichment analysis identified differential responses to fire blight infection at system-level functional pathways for ‘Gala’ and ‘Empire’. No significantly enriched terms were detected for ‘Empire’ DEGs whereas ‘Gala’ exhibited fifty significantly (*p* < 0.05) overrepresented pathways at 24 hpi (Fig. 4; Supporting file S2). Use of GO slim reduced the number of these significant terms to four broader categories associated with response to stress, cell cycle, response to stimulus, and nucleotide binding. The differential pathway response of ‘Gala’ and ‘Empire’ was also apparent at 48 hpi (Fig. 4; Supporting file S3). ‘Empire’ showed significant (*p* < 0.05) enrichment of pathways related to oxidoreductase activity and apoplast. In contrast, pathways related to defense response, ADP binding, and ubiquitin ligase complex were overrepresented in ‘Gala’. A greater number of significantly (*p* < 0.05) enriched pathways were identified at 72 hpi in both ‘Empire’ and ‘Gala’. ‘Empire’ displayed a total of 132 significantly (*p* < 0.05) enriched pathways, reduced to 34 after GO slim analysis (Fig. 4; Supporting file S4). ‘Gala’ showed 60 enriched functional classes that represented 21 broader pathways from GO slim analysis (Supporting file S4). Interestingly, many similar pathways were enriched in the two genotypes at 72 hpi. Genes associated with response to biotic stimuli, protein modification process, response to stress, kinase activity, transferase activity, DNA binding, and catalytic activity were prominent in both ‘Gala’ and ‘Empire’ at 72 hpi. In contrast, overrepresented GO terms related to distinct plant pathways were also observed in the two cultivars. ‘Empire’ had significant gene enrichments for pathways related to cell cycle, DNA metabolic process, motor activity, phosphorylase activity, cytoskeleton, and various extracellular and intracellular regions. ‘Gala’, in contrast, had fewer enriched GO terms related to cellular and metabolic processes. Overall, the pathway enrichment analysis emphasizes both genotypic and time-lapse transcriptome differences after fire blight infection in apple.

To gain further insights about the pathway functions, we separated the upregulated and downregulated genes at each time point to perform GO enrichment analysis (Supporting file S2, S3, S4). Pathways specific to cell cycle, biological regulation, motor activity, organelle, cytoskeleton, and pyrophosphatase activity were upregulated, whereas stress response, response to stimulus, cellular protein modification, nucleotide binding, and transcription factor activity pathways were downregulated during the initial stages (24 hpi) of fire blight infection. As the disease progresses, pathways related to defense, ADP binding, and vitamin binding were induced at 48 hpi. Oxidoreductase activity, apoplast, and ubiquitin ligase complex pathways showed repression at latter infection stages. At 72 hpi, pathways related to response to biotic stimuli, response to stress, cellular protein modification, kinase activity, catalytic activity, nucleotide binding activity, and transcription factor activity were commonly induced in the two genotypes. However, pathways specific to cell cycle, DNA metabolic process, regulation of metabolic process, protein binding, motor activity, pyrophosphatase activity, organelle, macromolecular complex etc. showed downregulation in ‘Empire’, while no significant GO term was associated with the downregulated genes in ‘Gala’ at 72 hpi (Supporting file S4).

### Co-expression modules associated with fire blight infection in apple

**Fig. 5:**
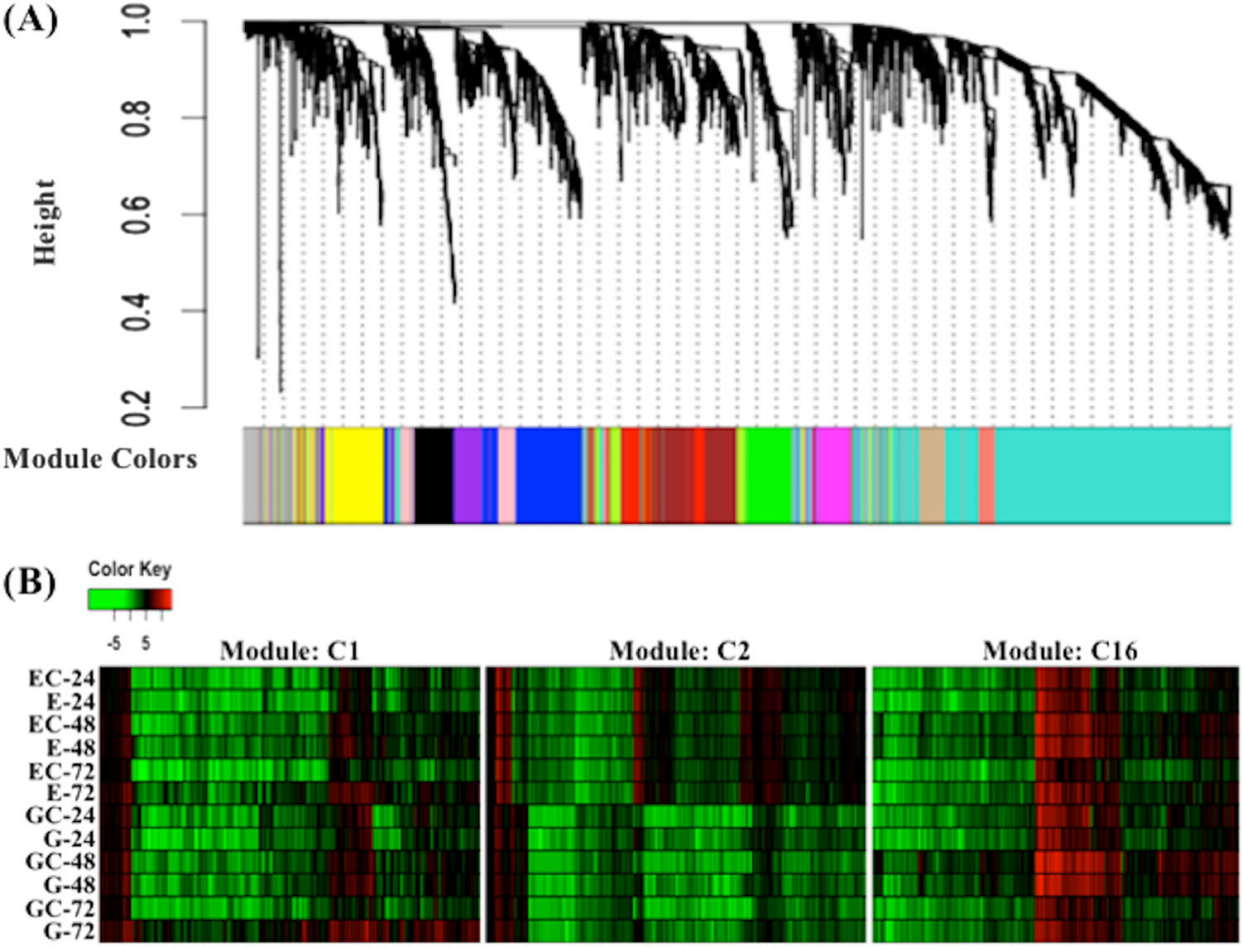
Weighted co-expression network analysis (WGCNA) of differentially expressed genes after fire blight infection in apple. **a** Gene co-expression modules identified from differentially expressed genes between ‘Empire’ and ‘Gala’ after fire blight infection using. **b** Gene expression patterns in three co-expression modules that specifically showed enrichment for defense response and response to biotic stimuli

Individual comparisons between control and treatment samples at 24, 48, and 72 hpi provided 4964 unique genes showing differential expression upon fire blight infection (Supporting file S5). We categorized these DEGs into distinct co-expression gene networks with weighted co-expression network analysis to explore gene expression patterns and regulatory networks related to fire blight. The normalized read count values for 4964 unique DE genes for each sample were used for cluster analysis. The co-expression analysis identified sixteen distinct gene clusters (Fig. 5a; Supporting file S5). The number of genes within each co-expression cluster exhibit considerable variation (Supporting file S6). For example, the largest cluster, C15, had 1773 (~35.7%) genes followed by the C2 (~9.9%, *n* = 495) cluster. Four clusters (C4, C9, C13, and C14) represents lowest number of genes ranging from 1.81% (*n* = 90) to 1.95 % (*n* = 97).

We further performed GO enrichment analysis to characterize functional classes associated with individual co-expression clusters (Supporting file S5). Four clusters (C5, C12, C13, and C14) did not show significant enrichment for any functional pathway. Some pathways were specifically identified in a single cluster. Among them, cluster C8 showed enrichment for ubiquitin ligase complex and ubiquitin-protein transferase activity. Similarly, clusters C1 and C2 had prominent enrichment of response to biotic stimulus and response to stimulus pathway genes. Such cluster-specific pathways were observed in almost all the co-expression clusters except C9. In contrast, several functional classes were observed in more than one cluster. For example, multiple clusters showed enrichment for ADP binding, nucleotide binding, catalytic activity, electron carrier activity, kinase activity, metabolic process, oxidation-reduction process, phosphorylation, and protein kinase activity. Overall, fire blight infection initiates system-level functional activity as revealed by the enrichment of broader functional GO terms. The presence of specific functional classes in more than one cluster indicates possible pathway interactions.

Three co-expression clusters (C1, C2, and C16) showed particular overrepresentation for the defense response, and response to abiotic stimuli (Fig. 5b). These clusters clearly represented different gene expression patterns across genotypes and treatments. For instance, genes within module C2 (*n* = 495) have distinct expression profiles in ‘Gala’ and ‘Empire’ across treatments. The genotypic differences between the remaining two clusters were less apparent, still they displayed time-lapse treatment effects within the two genotypes (Fig. 5b). A closer analysis of genes within each cluster using apple genome^34^ annotations revealed several functional candidates. All three clusters contained disease resistance proteins (TIR-NBS-LRR) and cytochrome P450 superfamily protein genes (Supporting file S6). Some pathogenesis related thaumatin superfamily proteins were also detected in module C1. In addition, several stress related transcription factors were present, including basic helix-loop-helix (bHLH), WRKY DNA-binding proteins, Zinc finger family proteins, AP2 domain and NAC domain proteins. The cytochrome P450 genes, disease-related proteins and transcriptional factors mostly showed upregulation during the later stages of fire blight infection (Supporting file S6).

### Gene expression validation

**Fig. 6:**
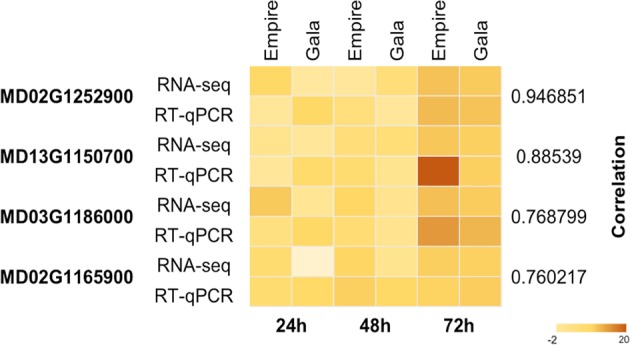
RT-qPCR validation of RNA sequencing results for ‘Empire’ and ‘Gala’ apple cultivars after fire blight infection. Four genes were selected for the confirmation of differentially expressed genes using the same RNA samples that were used for RNA sequencing. Three biological and three technical replicates were used for each sample and control. Pearson correlation between the gene expression levels measured using RT-qPCR and RNA-Seq was used for validation. The colors in the heat plot represents values for log2 fold change for each sample

We validated a set of differentially expressed genes with quantitative RT-PCR using a set of primers on DEGs from RNA-Seq analysis (Supporting file S7). The gene expression validation was performed using three biological replicates and three technical replicates of the control and infected samples at 24, 48, and 72 hpi. Expression values (log2 fold-change) from RNA-Seq results were compared to those derived from RT-qPCR analysis on the same RNA samples for both ‘Empire’ and ‘Gala’. Pearson correlation coefficients between log2 Fold change estimates from RNA-Seq and RT-qPCR ranged from 0.76 to 0.94 in all four genes for both ‘Empire’ and ‘Gala’ at respective time points. RT-qPCR fold-change values in ‘Empire’ and ‘Gala’ appear to generally agree with RNA-Seq, however the degree of regulation was generally higher in ‘Empire’ at 72 hpi, and lower in ‘Gala’ at 24 hpi (Fig. 6).

### Candidate genes in known fire blight resistance QTL regions

To identify the DEGs within previously described major effect fire blight QTLs^5,15^, we used QTL flanking intervals to extract apple genome coordinates and corresponding gene annotations for comparative analysis. A total of 241 DEGs were located across the four major effect QTL regions (Supporting file S8). About 48.5% and 31.1% of total DEGs in QTL regions were differentially expressed in ‘Empire’ or ‘Gala’, respectively, whereas 20.3% of genes showed differential expression in both apple cultivars. The number of DEGs ranged from 12 for LG 12 to 113 for LG 7. Analysis of functional annotations of DEGs in fire blight QTLs found candidate gene with previous association with disease resistance^28,30^. For example, receptor-like protein kinases and NB-ARC domain disease resistance genes were present in QTL regions from all the LGs (Supporting file S8). Another disease related gene from TIR-NBS leucine rich repeat family was identified in the fire blight QTL region on LG 7. Transcription factors such as WRKY domain, NAC domain, and C2H2 Zinc finger proteins were also among the DEGs in fire blight QTL regions.

## Discussion

A significant (*p*-value < 0.05) difference was seen for area under disease progress curve (AUDPC) between the cultivars ‘Empire’ and ‘Gala’ when infected with fire blight, demonstrating their differing levels of resistance. However, the necrotic length and percentage of infection were not significantly (*p*-value < 0.05) different in ‘Empire’ and ‘Gala’ until 4 days after infection, when ‘Empire’ started showing less disease than ‘Gala’. ‘Empire’ (‘McIntosh’ × ‘Red Delicious’) and ‘Gala’ (‘Kidd’s Orange Red’ × ‘Golden Delicious’) are economically important cultivars and have been referred to in the literature as moderately resistant and highly susceptible to *E. amylovora*, respectively^1,34–38^. Their distinct pedigrees might have contributed to these differences in disease progress and the gene expression patterns observed. ‘Red Delicious’ is considered resistant/tolerant to fire blight^39,40^ and may have passed this down to ‘Empire’. Although visible symptoms are not yet different at 72 hpi, the rate of disease progression started to differ between the two cultivars at this stage. ‘Empire’ seems to suppress or slow down bacterial progress more rapidly than ‘Gala’. Visual necrotic symptoms after 72 hpi are probably the result of earlier active processes, although subsequent transcriptome level changes might have contributed to these differences in two apple cultivars. The basis of varying susceptibility to fire blight in different apple cultivars might be the activation of different genetic mechanisms, even if the susceptibility levels vary from severe, mild to no differences at all^34^. Differences in gene expression patterns of ‘Empire’ and ‘Gala’ at three different time points of disease progression suggest that these cultivars deploy unique molecular mechanisms to overcome disease infection from *E. amylovora* strains, despite being of the same species. Wild malus species have been reported to have major resistance genes, but domesticated apples lack these major resistance genes, and have only minor resistance^5,7,10,15,27–30^. Comparing domesticated susceptible and moderately resistant apples would be expected to show more meaningful variations in gene expression in a highly heterozygous crop and could highlight some of the potential differences in the biological processes leading to resistance or susceptible responses. That is, when no major gene is present to provide strong resistance, a cultivar might employ a combination of strategies to avoid infection. A small step toward resistance might be more broadly applicable and biologically interesting than a major difference from a single R gene. The patterns of expression for defense-related genes can differ depending on the virulence of an *Erwinia* strain and apple cultivars used in a particular study. Nonetheless, the transcriptomic differences between ‘Empire’ and ‘Gala’ can highlight potential genes and molecular pathways to better understand the host-pathogen interaction in the *E. amylovora-Malus* pathosystem.

Differences in gene expression patterns between ‘Empire’ and ‘Gala’ were clear at 24 hpi and proceeded until 72 hpi. The transcriptional changes were more severe in ‘Gala’ at earlier stages of infection, but a contrasting trend was apparent in ‘Empire’ at 72 hpi, suggesting that the two cultivars have different molecular mechanisms for pathogen perception and control. These results also suggest that a time-course experiment is particularly informative for evaluating differential cultivar responses to fire blight infection. The early downregulation of genes related to stress response, response to stimulus, cellular protein modification, and transcription factor activity in ‘Gala’ might explain its inefficiency in restraining infection at earlier stages, which probably leads to its higher disease susceptibility. These defense pathways were induced as the infection proceeds. Although ‘Empire’ does not show gene enrichment for specific pathways at 24 hpi, induction of genes from leucine rich repeat family, cell division control, and MYB transcription factor family might provide some explanation for the defense against invading pathogens^41–43^. However, the role of early transcriptional changes to dictate the differential responses of ‘Empire’ and ‘Gala’ must be interpreted with caution, since no visible symptoms appear until 4 dai. With regard to the visible differences, it is very likely that transcriptional differences at 72 hpi contribute directly to the genotypic differences observed between the two apple cultivars. In particular, many pathogenesis-related genes, disease resistance protein (NBS-LRR) genes, and zinc finger domain genes displayed higher expression in ‘Empire’ than ‘Gala’ at 72 hpi. Metabolic processes are upregulated in ‘Empire’ at 72 h post infection, while several classes of organelle proteins are downregulated. Generally, it is considered that bacterial infection should induce downregulation of basic metabolic processes to save energy for resistance mechanisms. However, upregulation of metabolic processes during pathogen infection may also trigger signal cascades leading to host resistance^44^. Meanwhile, the targeting of host organelles by pathogenic bacteria to manipulate host functions and enable efficient infection has been suggested^45^. Modifying the behavior of organelles potentially targeted by bacteria could therefore be an effective resistance strategy. The many changes in expression of organelle proteins in ‘Empire’ could be an indication of its resistance, especially since we do not see these changes in expression in susceptible ‘Gala’. Exploration of gene functions within the activated pathways can provide more knowledge about bacterial invasion and multiplication inside the cells.

Enrichment analysis of DE genes showed several overrepresented pathways upon fire blight infection. The detection of genes for disease resistance and pathogenesis-related (PR) proteins agrees with the effective system-level response of the host plants upon infection. Members of disease resistance gene families have a known role in different biotic stresses and pathogen resistance^25,41^. Induction of these genes can explain the differential cultivar responses observed in ‘Gala’ and ‘Empire’ after fire blight infection. Some of the previously characterized resistance genes against fire blight belong to receptor-kinase repeat families^25,28,30,32,41^, and the disease resistance genes identified in this study can potentially lead to detection of novel sources of fire blight resistance. Enrichment analysis also identified specific functional categories related to general stress response. For instance, genes related to metabolic activity, cytochrome P450 gene family, protein modification process, kinase activity, transferase activity, and DNA binding show activation upon fire blight infection. These functional classes have been shown to respond under diverse stress categories^25,32,46–48^ and their significant enrichment in this study can be categorized as a general stress response. The activation of general stress pathways illustrates the system-level constraints imposed by bacterial infection on the host machinery.

Establishing marker-trait associations is one way to characterize genetic differences and gene-function relationships. Our study provides preliminary information for genome-wide characterization of disease related gene families in the apple genome to identify polymorphic markers and test association between any candidate genes and fire blight resistance. Several major effect quantitative trait loci (QTL) have been discovered against fire blight infection, mainly located on a few LGs on the apple genome^5,7,10,15,27–30^. All the QTLs, expect one on LG 7, have their original source of resistance from wild apple species. The genes and pathways identified in this study highlight potential sources of fire blight resistance from domesticated apples. Annotation of DEGs in the previously identified fire blight QTLs suggested several candidate genes related to known disease resistance gene classes. For example, members of NBS-LRRs, receptor kinases, and stress related transcription factor genes were present in fire blight QTL regions. These genes can be used to further explore the genetic mechanisms of fire blight resistance.

Co-expression gene networks provide multi-genic functional modules that can explain trait phenotypic differences to some extent^49–51^. The presence of distinct co-expression modules and functional enrichment of some modules in biotic stress and defense response suggests that significant variation at a gene expression level might explain the differential fire blight response of ‘Gala’ and ‘Empire’. Interestingly, these gene expression modules also contained different transcription factors (TFs), some of which have known association with biotic stress responses^42,52,53^. TFs can specifically bind to several downstream gene targets to regulate their expression and can explain the distinct transcriptome patterns observed in different co-expression modules. Thus, the TF regulated gene expression differences can explain some of the differential genotypic responses of ‘Gala’ and ‘Empire’ to fire blight. It will be interesting to explore whether these TFs have co-localized genomic positions with previously identified QTL for fire blight resistance.

In summary, fire blight infection activates a system-level response in apple. The differential transcriptome responses of ‘Gala’ and ‘Empire’ suggest that natural variation within domesticated apples can be tapped to understand host-pathogen interactions as well as to detect new sources of resistance against fire blight. The results presented here will increase the understanding of *E. amylovora-Malus* pathosystem and biological mechanisms associated with it.

## Materials and methods

### Plant material and *E. amylovora* inoculations

We obtained 1/4” M.7 rootstocks from Willamette Nurseries (Canby, OR, USA). ‘Empire’ (PI 588842) and ‘Gala’ (PI 392303) bud wood was collected from the US National Apple Collection at USDA-ARS Plant Genetic Resources Unit (PGRU) located in Geneva, NY. Trees were grafted and planted in D40H deepots (Stuewe and Sons, Tangent, OR) containing Cornell Potting mix (50 peatmoss:50 vermiculite with 6.2 kgm^−3^ lime, 1.25 kgm^−3^ superphosphate, and 0.62 kgm^−3^ calcium nitrate). Trees were allowed to acclimatize and grow in the greenhouse facility at Cornell AgriTech (Geneva, New York) at 24 °C, 50% RH, and natural light for 6 weeks.

Four-month-old grafted plants were used for inoculation. On the day of infection, actively growing grafted plants of each cultivar were moved to a growth chamber set at 24 °C, 75% RH, and 16 h light/8 h dark photoperiod. Eight biological replicates and two biological controls for each cultivar were inoculated by bisecting the youngest unfolded leaf with scissors dipped in bacterial suspension or 1× phosphate-buffered saline solution (1× PBS, pH 7.2) solution (controls) as described previously^24^ (Fig. 1). Inoculation was performed using the *E. amylovora* strain Ea2002A by reviving a frozen stock on King’s B medium (KB) incubated for 48 h at 28 °C. Bacterial cells were recovered in suspension using 1× PBS and adjusted to the concentration of 10^9^ CFU/ml on a SmartSpec Plus Spectrophotometer (Bio-Rad Laboratories, USA). Ea2002A, also referred as Ea265, was originally isolated from apples (*M*. × *domestica* ‘Jonathan’) in Ontario, Canada in 1980–81 (W.G. Bonn). This strain is highly aggressive against several apple cultivars^27,34^.

### Disease assessment

Lesion length (cm) was measured at 1, 2, 3, 4, 6, 9, 12, and 15 days after infection (dai). Percentage (%) of fire blight infection was determined as percentage lesion length (PLL): length of necrotic shoot divided by total shoot length multiplied by 100. PLL data points were further used to quantify Area Under Disease Progress Curve (AUDPC)^54^ as below:

$${\mathrm{AUDPC}} = \mathop {\sum}\nolimits_{i = 1}^{n - 1} {\left[ {\frac{{\left( {t_{i + 1} + t_i} \right)\left( {y_i + y_{i + 1}} \right)}}{2}} \right],}$$

Here, ‘*t*’ is time in days for each measurement, ‘*y*’ is the PLL at each measurement, and ‘*n*’ is the number of measurements. ANOVA and Student’s *t* tests were performed to assess the difference in traits between cultivars and among time points within a cultivar.

### Sample harvesting, RNA isolation, and sequencing

One strip (~1 cm in width) from each of the three expanded leaves located immediately below the inoculated leaf were cut and pooled together at 24, 48, and 72 h post infection in order to have enough material to extract RNA. Leaf tissues were collected from the same leaves and same plants to maintain uniformity between samples. Harvested plant tissues were immediately frozen in liquid nitrogen and stored at −80 °C to prevent RNA degradation.

Total RNA was isolated from infected and control samples using the Spectrum^TM^ Plant Total RNA Kit (Sigma-Aldrich, USA) according to the manufacturer’s protocol. Briefly, tissue was ground to a fine powder in liquid nitrogen using a mortar and pestle, and 100 mg of powder was transferred to a cold 2 ml microcentrifuge tube, then 500 µl of the Lysis Solution/2-ME Mixture was added to the tube. Samples were vortexed vigorously for at least 30 s and incubated at 56 °C for 5 min, then centrifuged for 3 min at 15,000 rpm. The lysate supernatant was transferred into a filtration column and centrifuged for 1 min at 15,000 rpm to remove residual debris. RNA binding step followed this as per manufacturer’s instructions. First, the clarified lysate was mixed with 250 µl of binding solution and vortexed briefly. The mixture was then pipetted into a binding column and centrifuged for 1 min at 15,000 rpm. The on-column DNase digestion step was performed for all samples. Consequently, after decanting the residual liquid retained in the collection tube, 300 µl of wash solution I was added to the binding column and centrifuged for 1 min at 15,000 rpm, then 80 µl of the DNase I: DNase digestion buffer (1:7) mixture was pipetted directly onto the center of the filter inside the binding column. Samples were incubated at room temperature for 15 min, and then washed (two times) with 500 µl of wash solution I at 15,000 rpm for 1 min in order to remove the digested DNA. Columns were centrifuged at maximum speed for 1 min to dry and then transferred to a new 2 ml microcentrifuge tube. Elution Solution (50 µl) was applied directly onto the center of the binding matrix inside the column. After 1 min, tubes were centrifuged at maximum speed for 1 min to elute. To increase RNA yields, the solution was pipetted back to the column and elution step was repeated. The quality and quantity of RNA were assessed by electrophoresis on 1% agarose gels and by a NanoDrop™ One/OneC Microvolume UV-Vis Spectrophotometer (Thermo Fisher Scientific, USA).

RNA library construction and sequencing were performed at Cornell University’s Genomics Facility at the Institute of Biotechnology (Ithaca, NY, USA; http://www.biotech.cornell.edu/brc/genomics-facility/services/library-construction-next-generation-sequencing). About 200 ng total RNA from two biological replicates from control samples and three biological replicates from the fire blight treated samples at each 24, 48, and 72 hpi was used to construct the 3′ RNA-Seq libraries using the QuantSeq 3′ mRNA-Seq Library Prep Kit FWD (Lexogen, USA) following the manufacturer’s instructions. Each sample was indexed with a unique adapter, and all libraries were pooled together and sequenced on one lane of an Illumina NextSeq 500 to generate 75 bp single-end sequence reads. Summary of raw RNA-Seq data is reported in Supporting file S1.

### RNA-Seq data processing and analysis

Raw RNA-Seq reads were processed to remove adaptor and low-quality sequences using Trimmomatic with default parameters^55^. The first 12 bases were then trimmed from each read and trimmed reads shorter than 36 bases were discarded. The remaining reads were aligned to the SILVA rRNA database^56^ using Bowtie^57^ allowing up to 3 mismatches, and those mapped to rRNA sequences were removed. The final high-quality cleaned reads from each library were aligned to the apple GDDH13 reference genome (v1.1)^58^ using STAR^59^ with the default parameters. Based on the alignments, raw count for each apple gene model in each library was derived by counting the total number of reads mapped the gene region from its midpoint to 500 bp downstream of its stop codon and then normalized to reads per million mapped reads (RPM).

### Differential gene expression and enrichment analysis

The read counts were used to perform differential gene expression analysis with DESeq2 v1.16.1^60^. Briefly, the control and treated samples were compared at each time-point within each cultivar using a generalized linear model to obtain log2Fold change differences and corresponding *p*-values for each gene model. Each treatment, time point, and cultivar were treated as distinct factors and the contrasts function in the DESeq2 program was used to extract the expression statistics for each comparison. To define a gene as differentially expressed, the DESeq2 output was filtered to retain genes that have multiple testing corrected *p*-value of <0.05 and a log2Fold threshold of 1.5. A gene having positive log2Fold values was considered as upregulated whereas negative log2Fold indicates a gene as downregulated upon bacterial treatment.

Gene ontology (GO) terms enriched in the upregulated and downregulated gene sets from individual time point comparisons of fire blight treatment were identified using Fisher’s exact test as implemented in agriGO v2.0^61^. Each gene set was compared against the full set of genes in the apple genome as background. Raw *p*-values were corrected for multiple testing using false discovery rate (FDR)^62^, and GO terms with FDR < 0.05 were identified as significantly enriched.

### Co-expression gene network analysis

The normalized read counts obtained from DESeq2 were used to construct co-expression gene networks with the weighted co-expression network analysis (WGCNA) package in R^63^. The differentially expressed genes between control and treatment samples at 24, 48, and 72 hpi from ‘Gala’ and ‘Empire’ were initially grouped together. The resulting dataset was filtered to remove redundant genes and to generate a set of unique gene IDs having differential expression in at least one of the sample comparisons. Corresponding normalized expression values were obtained for this set of unique genes to perform co-expression analysis. A one-step network building and module detection approach was used to build a co-expression gene network for fire blight infection in apple. WGCNA defines a network by connecting all variables in the dataset, which followed detection of modules with highly similar expression patterns. The initial step created an unsigned topological overlap matrix (TOM) to identify a threshold value for module detection. The network construction parameters included threshold power of 9, minimum module size equal to 30, and a branch merge cut height of 0.25. The resulting co-expression modules were visualized in Cytoscape v3.6.1^64^. For identification of disease- and stress-related modules, GO enrichment analysis was performed with the genes in each co-expression module using agriGO v2.0^61^.

### Candidate genes in known fire blight resistance QTL regions

We also performed a comparison of the positions of fire blight QTLs reported in previous literature^5,15^ with the differentially expressed genes in this study. To identify the DEGs, the QTL flanking markers were used to define the corresponding genomic regions on the apple GDDH13 reference genome (v1.1)^58^ in NCBI. The gene IDs within the QTL regions were compared with one from differential gene expression analysis of ‘Empire’ and ‘Gala. Corresponding gene annotations of the matching genes from QTL regions showing differential expression in ‘Empire’ and ‘Gala’ were used to define functional candidates for fire blight susceptibility.

### Quantitative real-time PCR (RT-qPCR)

A set of primers were designed from the RNA-Seq analysis to validate the expression of few genes. The gene expression was validated using three biological replicates and three technical replicates from the infected and control samples. First-strand cDNA synthesis was performed with 1 µg of total RNA using the Improm-II™ Reverse Transcription System (Promega, USA), according to the manufacturer’s instructions. Experimental RNA was combined with 1 µl of Oligo(dT)_15_ primer (0.5 µg/reaction) and nuclease-free water for a final volume of 7.5 µl per reaction. Samples were placed in 70 °C heat block for 5 min and then immediately chilled in ice-water for at least 5 min. The Reserve Transcription (RT) reaction mix was prepared by combining the following components of the ImProm-II™ Reverse Transcription System in a sterile 1.5 ml microcentrifuge tube on ice: 9.5 µl nuclease-free water, 6 µl ImProm-II™ 5X Reaction Buffer, 3.75 µl MgCl_2_ (1.5–8.0 mM), 1.5 µl dNTP (0.5 mM each dNTP), 0.75 µl Recombinant RNasin^®^ Ribonuclease Inhibitor, and 1 µl Reverse Transcriptase. After gentle homogenization, a 22.5 µl -aliquot of RT reaction mix was added to each 7.5 µl of RNA/primer mix, for a final RT reaction volume of 30 µl/sample. Samples were placed at 25 °C for 5 min and then incubated at 42 °C for 1 h. Reverse transcriptase was immediately inactivated by incubating samples at 70 °C for 15 min, and samples were diluted 10× before cDNA quantification. RT-qPCR was performed on a CFX96 Real Time System (Bio-Rad Laboratories, USA). A reaction mixture composed of 5 µl iTaq Universal SYBR Green Supermix (Bio-Rad Laboratories, USA), 1 µl of nuclease-free water, 1 µl of each forward and reverse desired primers (0.5 µM), and 2 µl of cDNA template to a total of 10 µl was used per reaction. Cycling conditions consisted of 95 °C for 3 min, followed by 45 cycles of 95 °C for 5 s and 60 °C for 30 s. A temperature gradient ranging from 60 °C to 95 °C with plate reads at every temperature increment of 0.5 °C was used to generate melting curve data. Four randomly selected candidate genes were used for RT-qPCR validation across three time points from two cultivars. Expression of candidate genes was normalized against the apple Elongation factor-1-alpha (EF1α) gene, one of the most stably expressed transcripts in apples^6,65^, and calculated using the formula 2^ΔΔCt^=2^[Ct(EF1α)–Ct(Gene)]^.

## Supplementary information

Fig. S1

Fig. S2

Fig. S3

Supporting file S1

Supporting file S2

Supporting file S3

Supporting file S4

Supporting file S5

Supporting file S6

Supporting file S7

Supporting file S8

Supporting figures and files

## Data Availability

Raw RNA-Seq data have been deposited in the NCBI Sequence Read Archive (SRA) under the accession number PRJNA489849.
