# Supplementary figures and images for: Differential gene regulatory pathways and co-expression networks associated with fire blight infection in apple (Malus × domestica)
Source: Hortic Res. 2019 Apr 6;6:35. doi: 10.1038/s41438-019-0120-z (PMC6441656; doi:10.1038/s41438-019-0120-z)

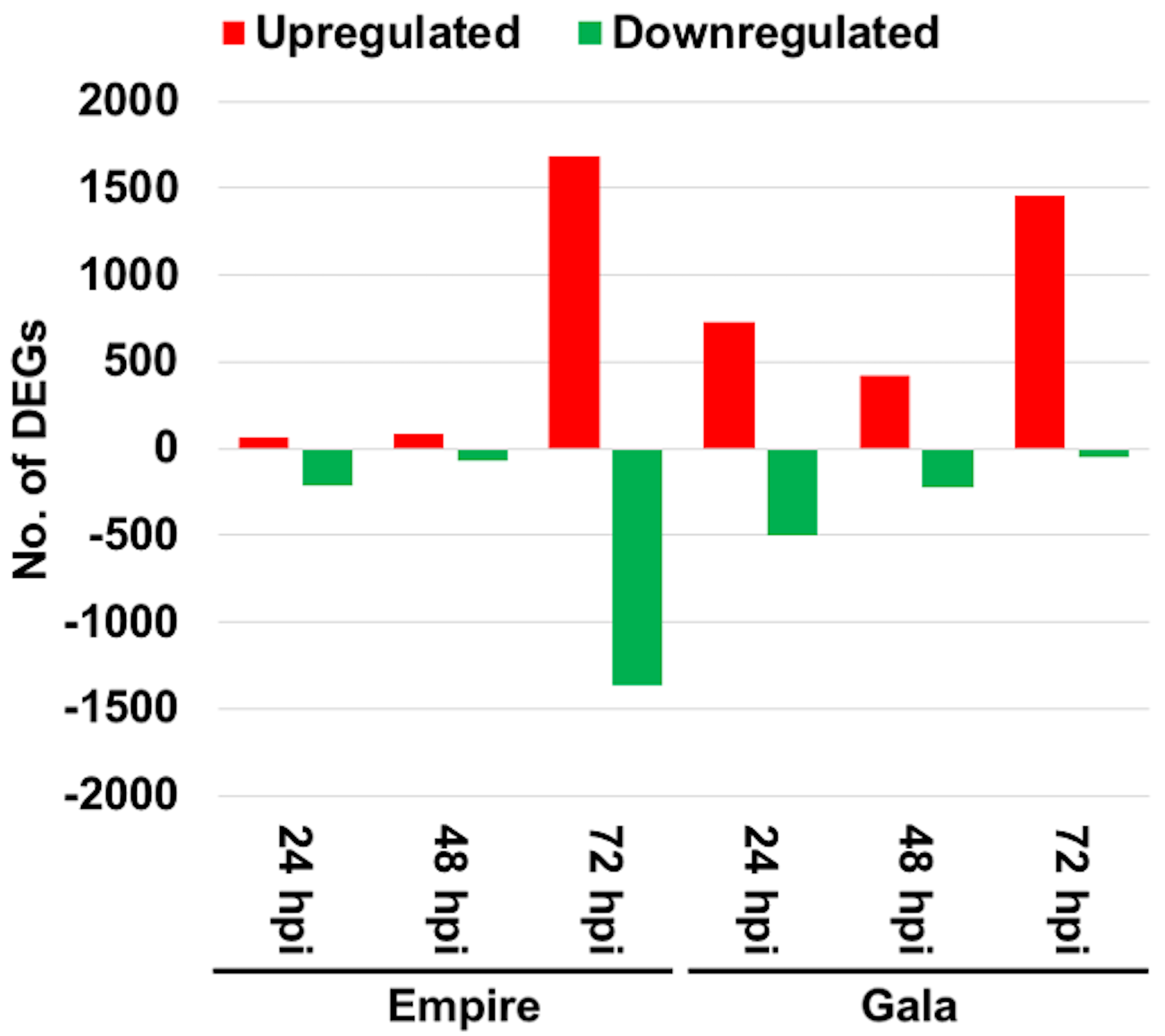

Supplement: Supplementary file 1 — Fig. S1 [file 41438_2019_120_MOESM1_ESM.png]

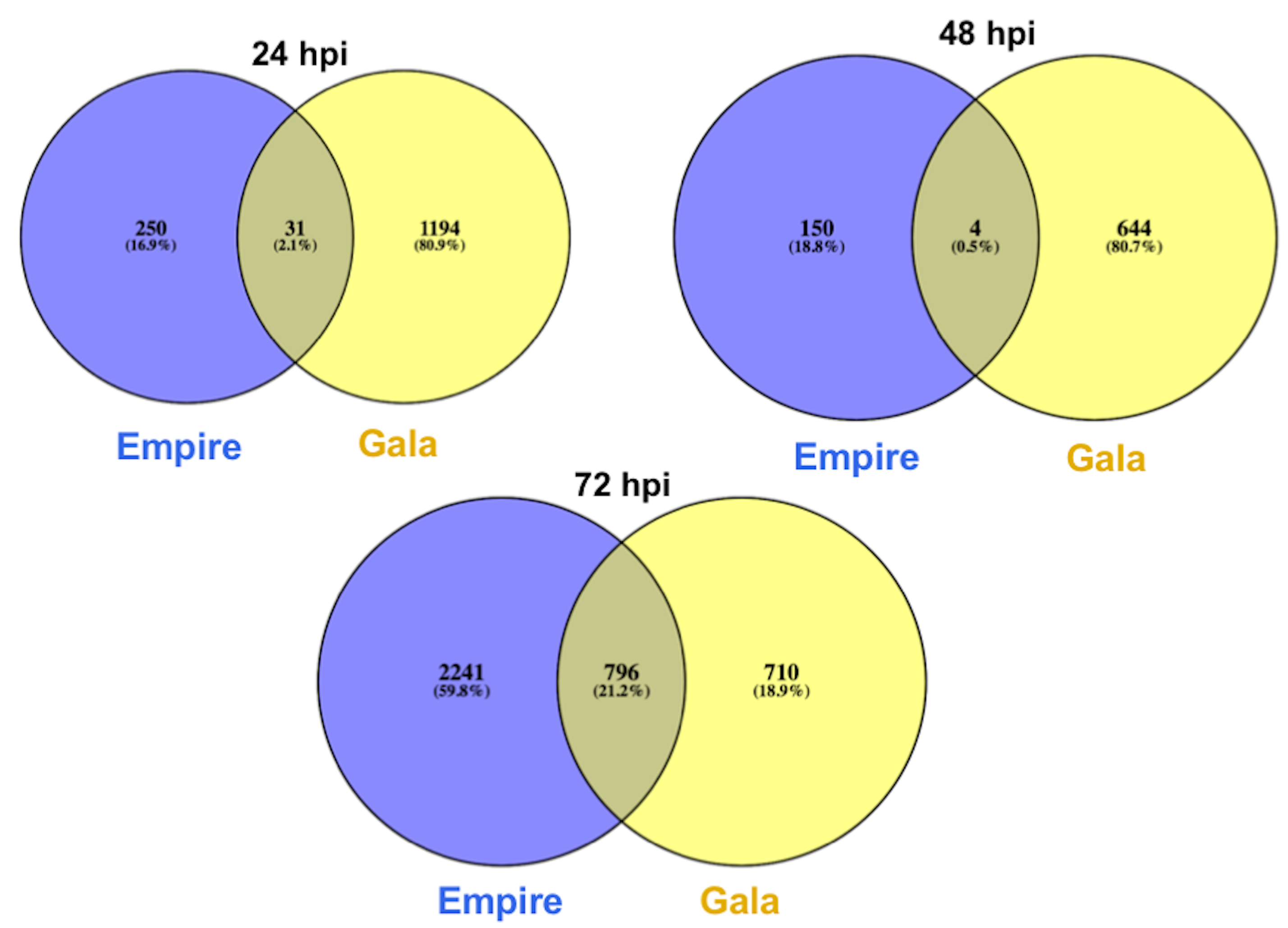

Supplement: Supplementary file 2 — Fig. S2 [file 41438_2019_120_MOESM2_ESM.png]

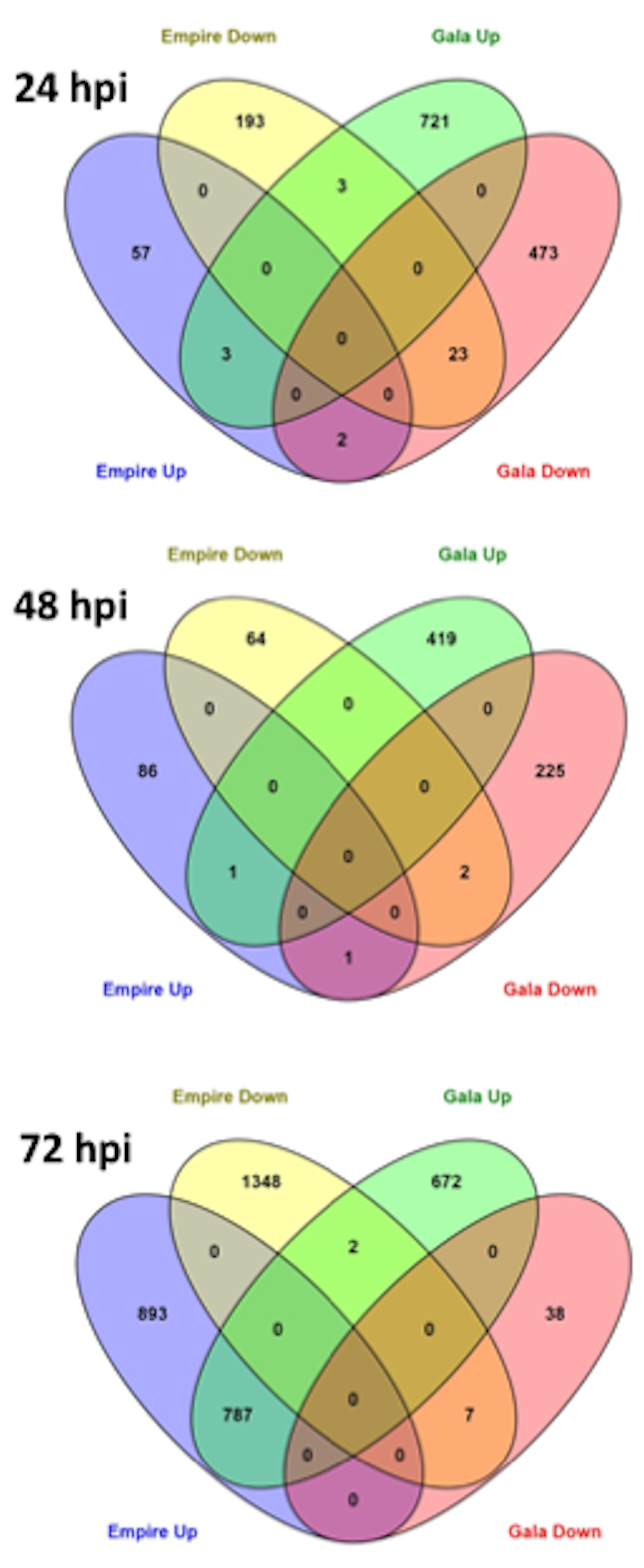

Supplement: Supplementary file 3 — Fig. S3 [file 41438_2019_120_MOESM3_ESM.png]
