# Supplementary material for: Differential gene regulatory pathways and co-expression networks associated with fire blight infection in apple (Malus × domestica)
Source: Hortic Res. 2019 Apr 6;6:35. doi: 10.1038/s41438-019-0120-z (PMC6441656; doi:10.1038/s41438-019-0120-z)
Supplement: Supplementary file 12 — Supporting figures and files [file 41438_2019_120_MOESM12_ESM.docx]

**SUPPORTING FIGURES AND FILES**

**Fig. S1** Number of significantly up- and downregulated genes in ‘Empire’ and ‘Gala’ apple cultivars at 24, 48, and 72 hours post inoculation with fire blight.

**Fig. S2** Number of DEGs uniquely expressed or shared between ‘Empire’ and ‘Gala’ apple cultivars at 24, 48, and 72 hours post inoculation with fire blight.

**Fig. S3** Number of upregulated and downregulated genes uniquely expressed or shared between ‘Empire’ and ‘Gala’ apple cultivars at 24, 48, and 72 hours post inoculation with fire blight.

**Supporting file S1**. Statistics of raw read cleaning and mapping for each sequenced sample from ‘Empire’ and ‘Gala’ apple cultivars after inoculation with fire blight and controls.

**Supporting file S2**. Gene ontology enrichment analysis of differentially expressed genes in ‘Empire’ and ‘Gala’ apple cultivars at 24 hours post inoculation with fire blight.

**Supporting file S3**. Gene ontology enrichment analysis of differentially expressed genes in ‘Empire’ and ‘Gala’ apple cultivars at 48 hours post inoculation with fire blight.

**Supporting file S4**. Gene ontology enrichment analysis of differentially expressed genes in ‘Empire’ and ‘Gala’ apple cultivars at 72 hours post inoculation with fire blight.

**Supporting file S5**. Summary of gene ontology (GO) enrichment analysis using genes specific to each co-expression module from WGCNA analysis for ‘Empire’ and ‘Gala’ apple cultivars after fire blight infection.

**Supporting file S6**. Functional annotation of genes within three modules (C1, C2, C16) that showed enrichment for defense response and response to biotic stimuli for ‘Empire’ and ‘Gala’ apple cultivars after fire blight infection.

**Supporting file S7**. Primers used to perform quantitative real time PCR to validate few differentially expressed genes for ‘Empire’ and ‘Gala’ apple cultivars after fire blight infection.

**Supporting file S8.** List of DEGs in the four major QTL regions that confer resistance against fire blight infection.
